# Longevity and stress in Caenorhabditis elegans

**DOI:** 10.18632/aging.100367

**Published:** 2011-08-28

**Authors:** Katherine I. Zhou, Zachary Pincus, Frank J. Slack

**Affiliations:** ^1^ Department of Molecular, Cellular and Developmental Biology, PO Box 208103, Yale University, New Haven, CT 06520

## Abstract

It has long been understood that many of the same manipulations that increase longevity in *Caenorhabditis elegans* also increase resistance to various acute stressors, and vice-versa; moreover these findings hold in more complex organisms as well. Nevertheless, the mechanistic relationship between these phenotypes remains unclear, and in many cases the overlap between stress resistance and longevity is inexact. Here we review the known connections between stress resistance and longevity, discuss instances in which these connections are absent, and summarize the theoretical explanations that have been posited for these phenomena.

## INTRODUCTION

*Caenorhabditis elegans* is an informative and convenient model for aging and stress studies. It was the first multicellular organism to have its complete genome sequenced [1], and its small size, transparency, and short life cycle further simplify studies of aging, genetics, and whole-body stress [2-4]. Often, manipulations that increase *C. elegans* longevity also increase stress resistance; conversely, mobilizing stress responses in the nematode can prolong lifespan. Many of the classic longevity mutations in *C. elegans*, including those in the insulin/insulin-like growth factor (IGF-1) receptor signaling pathway [5-7] and those involved in determining the rate of mitochondrial respiration [8, 9] or of feeding [10], also confer resistance to various stressors such as high temperature [11, 12], UV irradiation [13], reactive oxygen species [14-17], or pathogens [18]. Other lifespan-extending manipulations, such as dietary restriction or germline ablation, also increase resistance to thermal and oxidative stress [19-21]. Further, low-level stressors on the order of 20-25% of the minimum toxic dose often enhance future stress resistance and increase longevity, through a process termed stress-induced hormesis [22-26]. While pre-treatment with oxidative or thermal stress has been shown to increase stress resistance and lifespan, not all forms of stress produce these effects ionizing radiation does not consistently induce a hormetic response in *C. elegans* [22, 27].

In this work, we first review several well-studied stressors in *C. elegans* and downstream effectors of the responses to those stressors, with an eye toward the effects of those effectors on longevity. Next, we examine common genes, pathways, and physiological processes that when manipulated extend lifespan, and the relationship of these manipulations to stress responses. The longevity and stress-response phenotypes of the genes discussed are summarized in Table 1 for convenient reference. Finally, we attempt to integrate these findings with evolutionary perspectives on the genetic control of longevity.

**Table 1:** Genes involved in lifespan and stress-resistance

Gene	Homologues	Principal Pathway(s)	Effect of gene suppression on
lifespan	stress tolerance
*eat-2*	non-alpha-subunits of nicotinic acetylcholine receptor (nAChR)	pharyngeal pumping	increased [174]	increased thermotolerance [142, 174]
*pep-2*	oligopeptide transporter	peptide uptake	none [180]	increased resistance to thermal and oxidative stress [180]
*daf-2*	mammalian insulin and insulin growth factor (IGF-1) receptors	IIS	increased [91]	increased resistance to thermal [12], oxidative [92], heavy-metal [72], and pathogenic stress [18]
*age-1*	p11, catalytic subunit of human phosphatidylinositol 3-kinase (PI3K)	IIS	increased [12, 34]	increased resistance to thermal [12], oxidative stress [34], heavy-metal [72], and pathogenic stress [18]
*akt-1*	mammalian S/T kinase Akt/PKB	IIS	weakly increased [96, 97]	weakly increases resistance to thermal and oxidative stress [81]
*akt-2*	mammalian S/T kinase Akt/PKB	IIS	weakly increased [96, 97]	weakly increases resistance to thermal and oxidative stress [81]
*sgk-1*	serum- and glucocorticoid-inducible kinase SGK	IIS	increased [81]	increased resistance to thermal and oxidative stress [81]
*daf-18*	human tumor suppressor PTEN	IIS	decreased [5, 98]	decreased resistance to oxidative stress [126]
*vab-1*	Eph receptor tyrosine kinase	IIS	increased [101]	N.D.
*daf-16*	Forkhead box proteins class O (FOXO)	IIS	decreased [88]	decreased thermotolerance[104]; no effect on resistance to UV, oxidative, or pathogenic stress [18, 103]
*sir-2*	yeast Sir2, mammalian SIRT	IIS	decreased [87]	decreased resistance to thermal, oxidative, and UV stress [87]
*jnk-1*	mammalian c-Jun N-terminal kinase (JNK)	IIS	decreased [119]	decreased resistance to thermal stress [119]
*lin-14*	(putative transcription factor)	IIS	increased [89]	increased resistance to thermal stress [89]
*hsf-1*	heat-shock transcription factor	heat-shock response, IIS	decreased [88]	decreased resistance to thermal [211], oxidative [212], and pathogenic stress [105]
*smk-1*	mammalian suppressor of MEK1 null (SMEK)	IIS	weakly decreased [90]	decreased resistance to pathogenic stress [90]
*hcf-1*	mammalian host cell factor 1 (HCF-1)	IIS	increased [121]	increased resistance to oxidative and heavy-metal stress; no effect on resistance to thermal stress [121]
*cep-1*	tumor suppressor p53	IIS	increased [125]	no effect on resistance to thermal, oxidative, UV, or pathogenic stress [125]
*smg-1*	S/T kinase in nonsense mediated mRNA decay	IIS	increased [126]	increased resistance to oxidative stress [126]
*skn-1*	transcription factor	IIS	decreased [133]	decreased resistance to oxidative stress [133]
*let-363*	S/T kinase target of rapamycin (TOR)	TOR	increased [143]	increased thermotolerance [142]
*daf-15*	Regulated Associated Protein of TOR (Raptor)	TOR	increased [140]	N.D.
*ife-2*	eukaryotic initiation factor 4E (eIF4E)	mRNA translation	increased [144]	increased resistance to thermal [142] and oxidative stress [144]
*bec-1*	yeast and mammalian autophagy genes Apg6/Vps30p/beclin1	autophagy	weakly decreased [94]	N.D.
*isp-1*	iron sulfur protein of complex III in ETC	mitochondrial respiration	increased [8, 9]	increased resistance to oxidative stress [8, 9]
*lrs-2*	mitochondrial leucyl-tRNA synthetase	mitochondrial	increased [152]	decreased resistance to oxidative stress [152]
*clk-1*	hydroxylase for ubiquinone synthesis	mitochondrial respiration	increased [157]	*clk-1* mutation increased oxidative stress resistance in *daf-2* mutants [92]
*mev-1*	subunit of complex II	mitochondrial respiration	decreased [104, 159]	decreased resistance to oxidative stress [160]
*cchl-1*	cytochrome c heme lyase	mitochondrial respiration	increased [152]	no effect on resistance to oxidative stress [152]
*sod-1*	cytosolic Cu/ZnSOD	antioxidant enzymes	weakly decreased [164]	decreased resistance to oxidative stress [164, 165]
*sod-2*	mitochondrial MnSOD	antioxidant enzymes	slightly increased [166]	decreased resistance to oxidative stress [164, 166]
*sod-3*	mitochondrial MnSOD	antioxidant enzymes	no effect [166]	no effect [165]
*sod-4*	(predicted) extracellular Cu/ZnSOD	antioxidant enzymes	no effect [166]	no effect [165]
*sod-5*	cytosolic Cu/ZnSOD	antioxidant enzymes	no effect [166]	no effect [165]
*ctl-1*	cytoplasmic catalase	antioxidant enzymes	no effect [213]	N.D.
*ctl-2*	peroxisomal catalase and peroxidase	antioxidant enzymes	decreased [213]	N.D.
*mtl-1*	metallothionein	antioxidant enzymes	N.D.	decreased resistance to cadmium stress [70, 214]
*glp-1*	Notch family receptors (N-glycosylated transmembrane protein)	germline	increased [21]	increased resistance to pathogenic stress [215]
*mes-1*	receptor tyrosine kinase	germline	increased [21]	N.D.

## STRESS RESPONSES IN *C. ELEGANS*

### Oxidative stress

Endogenous or exogenous reactive oxygen species (ROS) damage to protein, lipid, and nucleic acid components of the cell is known generically as oxidative stress. Unavoidably, during mitochondrial respiration, 0.4-4% of the consumed oxygen is transformed into various reactive byproducts, especially superoxide (×O_2_^−^) [28]. Superoxide reacts with iron- sulfur clusters to release iron, or is converted to hydrogen peroxide (H_2_O_2_), which reacts with free iron to make hydroxyl radicals (OH×); these free radicals can then oxidize macromolecules, impairing their function [28, 29]. To protect against these species, nearly all cells, both prokaryotic and eukaryotic, use the enzymes superoxide dismutase (SOD), catalase, and glutathione peroxidase, which convert superoxide and other ROS into less-reactive forms (often eventually water) [30-33].

*C. elegans* can be experimentally subjected to exogenous oxidative stress by exposure to compounds such as *tert*-butylhydroperoxide, arsenite, paraquat, and juglone. High concentrations of any of these greatly reduces *C. elegans* lifespan [14, 17, 34-37]. Conversely, antioxidant compounds can be used to experimentally reduce the risks posed by endogenous and exogenous oxidative stressors. Nematodes have been found to live longer when treated with vitamin E [38], α-lipoic acid [39], resveratrol [40], or the catalytic antioxidants Euk-8 and Euk-134 [41], and new studies continue to show the beneficial role of various antioxidants on longevity [42-45]. However, other researchers have been unable to reproduce the extension of lifespan by Euk-8 [46], and the results for vitamin E have also been inconsistent [36]. These inconsistencies may be due to differences in dosage or culture conditions – for instance, it has been suggested that early results showing that certain antioxidants extend *C. elegans* lifespan may have been caused by dietary restriction instead [36, 46, 47]. Even for antioxidants consistently found to extend lifespan, it is not always clear whether the mode of action is in fact antioxidant activity: since α-lipoic acid and the vitamin E derivative trolox also increase thermotolerance, it has been suggested that they increase longevity by inducing a stress response [36, 39, 47]. Furthermore, other antioxidant molecules, such as N-acetylcysteine and vitamin C, do not appear to extend lifespan [36]. Subjecting *C. elegans* to low amounts of oxidative stress can also induce longer lifespan and heightened stress resistance via hormesis: nematodes pre-treated with hyperbaric oxygen or juglone subsequently exhibit increased resistance to both oxidative stressors; in addition, animals pre-treated with hyperbaric oxygen have a 20% longer life expectancy [22]. Oxygen pre-treatment has also been shown to increase X-radiation resistance in *C. elegans* [48].

Various indicators have been used to assess the extent of oxidative damage during aging. One common indicator is protein carbonyl content [16, 38, 49]; another is an autofluorescent species known as lipofuscin. This “age pigment” accumulates over time in many different species, including *C. elegans* [50], and mammalian studies suggest that its accumulation may be due to oxidative stress [51-54]. Similarly, some studies have shown that mutant animals with increased mitochondrial ROS damage have faster accumulation of fluorescent pigments, including lipofuscin [55, 56]. Other studies did not replicate these observations, however, suggesting that mitochondrial oxidative stress is not solely responsible for the accumulation of endogenous fluorescent compounds such as lipofuscin and advanced glycation end-products [57]. These inconsistent results may be due to disparities between pigment-measurement protocols [19, 57].

### Thermal stress

Laboratory *C. elegans* are typically grown at 15-25°C; temperatures of 30-35°C constitute stressors. In response to high-temperature conditions, prokaryotes and eukaryotes alike upregulate heat-shock proteins (HSPs). Many of these proteins function as molecular chaperones, helping unfolded and misfolded proteins assume the correct conformation. HSP-4 and HSP-16 are two such proteins that accumulate in response to thermal stress in *C. elegans* [58-60]. The *C. elegans* heat-shock factor (HSF) is a transcription factor thought to regulate the response to thermal stress, and *hsf-1*(RNAi) has been shown to accelerate aging [61]. When first-larval stage (L1) or early L2 *C. elegans* larvae are subjected to thermal stress or food limitation, they enter an alternative, stress-resistant, growth-arrested L3 state called dauer [62]. Dauers have lower metabolic rate, are resistant to oxidative stress, and express higher levels of antioxidant enzymes and HSPs [14, 34, 62, 63]. Short exposure of adult wild-type *C. elegans* to high temperatures does not induce entry into a state of diapause, but instead can cause significant decrease in fertility [11]. Moreover, several hours of exposure to high temperatures can kill nematodes. As such, *C. elegans* thermotolerance is often assessed by measuring fertility or survival rate [11]. Treatment with certain antioxidant compounds, such as α-lipoic acid or trolox, increases both thermotolerance and lifespan in *C. elegans* [39]. Though these antioxidant treatments do not affect total fertility, in some cases they cause delayed or temporarily reduced fertility [39].

Thermal stress also has demonstrated hormetic effects.*C. elegans* exposed to 35°C for up to two hours exhibit greater tolerance to subsequent thermal stress and also extended life span [12, 22, 22, 64, 65]. While a single heat shock early in life gives rise to a 20% longer lifespan, multiple mild heat shocks throughout the *C. elegans* lifetime increase its lifespan by about 50% [24, 58, 66]. Furthermore, a single shock seems to increase lifespan without slowing aging, whereas multiple heat shocks both increase lifespan and slow the process of aging [24].

### Heavy-metal stress

Trace levels of nonessential heavy metals such as cadmium, arsenic, mercury, and lead, as well as supraoptimal levels of the essential heavy metals copper and zinc, are toxic to cells. The mechanism behind heavy-metal toxicity is not well understood, but it is thought that heavy-metal ions cause damage to the cell by inactivating and denaturing proteins, and by promoting the formation of reactive oxygen species [67-69]. The thiol tripeptide glutathione, a class of cysteine-rich proteins called metallothioneins, and other larger metal-binding proteins protect the cell from heavy-metal stress by chelating and sequestering metal ions [67, 69, 70]. Heavy-metal stress also activates the expression of cell repair mechanisms, including heat-shock proteins [70]. The KGB-1 and PMK-1 mitogen-activated protein kinase (MAPK) pathways also function in the heavy-metal stress response, and the MAPK phosphatase (MKP) VHP-1 modulates this response by downregulating these two pathways [71]. Other genes that contribute to the heavy-metal stress tolerance of *C. elegans* have been identified by DNA microarray and RNAi studies, including the metallothionein-encoding genes *mtl-1* and *mtl-2* [70], the nuclear localized metal responsive proteins NUMR-1 and NUMR-2 [69, 70], and *hmt-1*, which encodes an ATP-binding cassette (ABC) transporter likely involved in cadmium sequestration [67]. Heavy-metal stress response may be regulated by the transcription factors DAF-16 and SKN-1, since SKN-1 activates metal detoxification genes including *hmt-1* when *C. elegans* is exposed to the metalloid sodium arsenite [37], and DAF-16 regulates the expression of *mtl-1* [72]. Moreover, putative binding sites for DAF-16 and SKN-1 have been found upstream of *numr-1* and *numr-2* [69]. Since DAF-16 and SKN-1 also play roles in the increased lifespan of longevity mutants, these transcription factors form a potential link between heavy-metal stress resistance and *C. elegans* longevity pathways. Consistent with this possibility, the long-lived mutants *daf-2* and *age-1* are resistant to heavy-metal stress, and *daf-2* animals have elevated levels of *mtl-1* mRNA [72].

## GENES AND PHYSIOLOGICAL PROCESSES THAT CONNECT LONGEVITY AND STRESS RESISTANCE IN *C. ELEGANS*

### Insulin/IGF-1 Signaling

The insulin/IGF-1-like signaling (IIS) pathway is an evolutionarily conserved endocrine signaling pathway that regulates food storage and growth [73]. Across taxa, reductions in insulin signaling and/or responsiveness are used as organismal signals for various types of stress – for example, IIS is involved in the entry into dauer in *C. elegans*. IIS pathway activity also influences lifespan in multiple different organisms, including yeast, nematodes, flies, and mammals [74-76], and it has been proposed that the regulation of longevity via the IIS pathway may have evolved to enable animals to survive harsh, stressful conditions [75]. In *C. elegans*, the gene *daf-2* encodes a homolog of the mammalian insulin and insulin growth factor (IGF-1) receptors [7]. When ligand-bound, DAF-2 triggers the IIS pathway [77] by activating AGE-1, a homolog of p11, the catalytic subunit of human phosphatidylinositol 3-kinase (PI3K) [6]. (Of note: allelic variation in PI3K appears to affect human longevity, as determined by comparing the representation of genotypes in younger and older age groups [76, 78]). Once activated by DAF-2, AGE-1 catalyzes the formation of the second messenger phosphatidylinositol-3,4,5-triphosphate (PIP3), which in turn binds and activates AKT-1 and AKT-2, homologs of the mammalian serine/threonine kinase Akt/PKB [6, 79, 80]. Activated AKT proteins form a complex with SGK-1, a homolog of the serum- and glucocorticoid-inducible kinase SGK. All three kinases in this complex can directly phosphorylate the FOXO-family transcription factor DAF-16, constraining it to the cytoplasm [81-83]. Absent IIS activity, dephosphorylated DAF-16 enters the nucleus and regulates the transcription of a variety of genes regulating lifespan, dauer formation, stress resistance, development, and metabolism, among others [84]. IIS activity is antagonized by DAF-18, a homolog of the human tumor suppressor PTEN. DAF-18 limits AKT-1 and AKT-2 activity by dephosphorylating PIP3 [5, 85]. In the nucleus, DAF-16 is further regulated by several different proteins, including the transcription factor HSF-1 and the SMEK ortholog SMK-1 [86-90].

Certain mutant *daf-2* alleles confer two-fold increased longevity and resistance to various stresses, including heat shock, oxidative stress, heavy-metal stress, and infection [12, 18, 72, 91, 92]. These mutants express higher levels of antioxidant enzymes such as catalase and SOD [92]. Additionally, *daf-2* mutants often constitutively enter the dauer state, and some alleles also confer other pleiotropic traits including reduced adult motility, abnormal adult morphology, and reduced brood size [93]. Mutants in *daf-2* also have an increase in autophagy, a catabolic process through which proteins and organelles are delivered to lysosomes for degradation. The autophagy gene *bec-1*, the homolog of mammalian beclin 1, is required for the increased lifespan of *daf-2* mutant animals [94], suggesting that autophagy is an essential lifespan-prolonging component of the stress responses typically held in check by IIS activity.

Many mutations in IIS pathway genes downstream of *daf-2* also modulate *C. elegans* lifespan and stress resistance. *age-1(hx546)* animals, which have a reduction-of-function point mutation in the *age-1* gene, exhibit a 40-110% extension in mean lifespan, and higher tolerance to thermal stress [12, 14, 15, 34, 95]. Furthermore, the relative activity of catalase and SOD in these animals, as compared to wild-type, increases with age in a manner that correlates with an age-dependent increase in the mutant animals' relative stress resistance [14, 34, 95]. Null mutants for *age-1*, in which the AGE-1 protein is truncated upstream of its kinase domain, have a mean lifespan about ten times that of wild-type animals [15]. Compared with the weaker *age-1(hx546)* allele, these animals are significantly more tolerant to oxidative stress, but also slightly less tolerant to thermal stress [15]. It is also worth noting that classic lifespan-extending mutants, including *daf-2* and *age-1* nematodes, exhibit heightened resistance to various bacterial pathogens [18].

Mutations in *akt-1* and *akt-2* have a surprisingly small effect on *C. elegans* lifespan. *akt-1(mg306)* mutants, which have a point mutation in the *akt-1* gene, live only around 10% longer than wild-type [96, 97]. The mutants *akt-1(ok525)* and *akt-2(ok393)* lack most of the kinase domain necessary for Akt/PKB activity, yet do not exhibit extended lifespan or increased stress resistance, while *akt-2(ok393)* mutants subjected to *akt-1*(RNAi) have only a 19% lifespan extension as well as a weak increase in resistance against high temperatures and oxidative stress [79, 81]. On the other hand, *sgk-1*(RNAi) significantly increases lifespan as well as resistance to heat and oxidative stress, suggesting that SGK-1 may be more important for the regulation of stress response and lifespan, whereas AKT-1 and AKT-2 are more important in regulating dauer formation [81].

Mutations in *daf-18*, an inhibitor of the IIS pathway, decrease *C. elegans* lifespan [5, 98]. A mutation in *daf-18* partially suppresses the increased lifespan of *daf-2* animals [98], and *daf-18* also necessary for the nuclear localization of *daf-16* in *daf-2* animals [99].

Interestingly, *daf-18* mutants subjected to hormetic levels of thermal stress do not have longer lifespans [100]. Though other *C. elegans* genes have been found to be required for various forms of hormesis (the radiation-sensitive mutants *rad-1* and *rad-2* mutants do not exhibit increased resistance to X-ray irradiation subsequent to pre-treatment with oxidative stress [48]), *daf-18* appears to be the only such gene that does not also directly disrupt stress response mechanisms. Further, it has been proposed that VAB-1, an Eph receptor tyrosine kinase, diminishes DAF-18 activity by phosphorylation [101]. Both *vab-1* mutants and *C. elegans* overexpressing *daf-18* live longer and are more sensitive to dauer conditions than wild-type [101], but the potential regulation of DAF-18 by VAB-1 remains to be investigated in the context of lifespan extension and stress tolerance.

In all cases mentioned above, the increased lifespan and stress resistance of IIS pathway mutants is dependent on *daf-16* [15, 81, 93, 98]. Null mutants in *daf-16* have shortened lifespan, and their germ cells are hypersensitive to ionizing radiation [88, 102]. Interestingly, *daf-16* mutants exhibit similar levels of resistance to UV, oxidative stress, and pathogenic stress as compared to wild-type animals [18, 103], although *daf-16* animals are less thermotolerant than wild-type [104], and *C. elegans* which overexpress *daf-16* are more resistant to *P. aeruginosa* [105].

Since DAF-16 is thought to be the main target of the IIS pathway, it has long been of interest to identify the genes which it regulates. The stress-response genes *sod-3*, which codes for a mitochondrial MnSOD, and *mtl-1*, which codes for a metallothionein homolog, were among the first clear direct DAF-16 targets [72, 92]. Since then, a series of studies has revealed many antioxidant, metabolic, heat-shock, and antibacterial genes to be targeted by DAF-16 [106-109]. Furthermore, a DNA microarray study aimed at finding DAF-16 targets also uncovered two putative DAF-16 binding sites based on the over-representation of these sequences upstream of target genes [107]. One of these sequences has also been shown to be bound by DAF-16 *in vitro* [110].

In addition to IIS, other longevity pathways also converge on DAF-16. One such pathway involves the sirtuins, a highly conserved family of NAD^+^-dependent protein deacetylases that includes SIR-2.1 in *C. elegans*, Sir2 in yeast, and the SIRT proteins in mammals. Among other functions, sirtuins deacetylate histones, thereby playing a role in the regulation of chromatin state [111-113]. The sirtuins are known to regulate stress response and cell senescence – for instance, Sir2α in yeast represses stress-induced, p53-mediated apoptosis [40, 114-116]. In *C. elegans*, SIR-2.1 activates DAF-16, and *sir-2.1* mutants are short-lived and stress sensitive, while overexpression of *sir-2.1* increases lifespan by 50% in a *daf-16* dependent manner [86, 87]. Following heat shock, SIR-2.1, which is localized to the nucleus, physically interacts with DAF-16, and this interaction depends on PAR-5 and FTT-2, which are homologs of 14-3-3 proteins, small acidic proteins that bind phosphoserine and phosphothreonine residues in particular sequence contexts [117]. *Sir-2.1* is however not required for the longevity of IIS pathway mutants, leading to the suggestion that SIR-2.1 and the 14-3-3-like proteins act in a stress-dependent manner to activate DAF-16 and increase lifespan, which is nevertheless independent of IIS [117].

Another pathway converging on DAF-16 involves a member of the JNK family. The mammalian mitogen-activated c-Jun N-terminal kinases (JNKs) can be activated by cytokines or environmental stress and are known to regulate processes including development and cell survival [118]. JNK-1, the *C. elegans* homolog of mammalian JNK, is thought to regulate lifespan in parallel to the IIS pathway, by regulating the subcellular localization of DAF-16 [119]. Whereas phosphorylation of DAF-16 by the AKT and SGK kinases constrains it to the cytoplasm, phosphorylation of DAF-16 by JNK-1 causes it to translocate to the nucleus [119]. Consistent with this observation, *jnk-1* mutants have shorter lifespan than wild-type, and *C. elegans* overexpressing *jnk-1* exhibit extended lifespan in a manner independent of IIS but dependent on *daf-16* [119]. The putative transcription factor LIN-14, which is required for normal lifespan (see below), has also been found to regulate DAF-16, though the mechanism of this regulation remains unknown [89].

Nuclear localization of DAF-16 is neither necessary nor sufficient for lifespan extension [99]: many additional regulators act on this transcription factor once it has translocated to the nucleus, and translocation is not increased in certain *daf-16*-dependent longevity mutants. When mutations are introduced at the AKT phosphorylation sites on DAF-16, the transcription factor localizes to the nucleus, but the animals expressing this mutant DAF-16 do not constitutively become dauers and do not have extended lifespan [83]. The transcription factor HSF-1, which regulates the heat-shock response in *C. elegans*, is thought to act together in the nucleus with DAF-16 to activate the transcription of certain genes, including small heat-shock proteins [88]. Similarly, the SMEK ortholog SMK-1 appears to regulate the transcriptional specificity of DAF-16 already localized to the nucleus [90]. The mammalian protein SMEK1 is phosphorylated in response to stress and is involved in the regulation of glucose metabolism under varying conditions, such as fasting [120]. It is thus conceivable that SMK-1 in *C. elegans* also modulates DAF-16 specificity as a response to certain stressors. In fact, the *smk-1* gene in *C. elegans* is required for *daf-2* mutants to exhibit extended lifespan, as well as resistance to pathogenic, UV, and oxidative stress. However, reduced activity of *smk-1* does not affect the thermotolerance of *daf-2* nematodes [90]. In addition, the *C. elegans* homolog of the highly conserved protein host cell factor 1 (HCF-1) is localized to the nucleus and physically associates with DAF-16 [121, 122]. Mammalian HCF-1 plays key roles in cell-cycle progression and has been shown to act primarily by binding to transcription factors and by assembling protein complexes [121, 122]. Mutations in the *C. elegans* gene *hcf-1* result in a 40% increase in lifespan and greater resistance to oxidative stress, but not to heat stress [121]. It has been proposed that HCF-1 negatively regulates DAF-16 by forming a complex with DAF-16 in the nucleus and thus limiting access of DAF-16 to its target promoters [121].

Conversely, some results have indicated that DAF-16 function may not be limited to the nucleus [123]. First, though the longevity of *age-1* mutants depends on *daf-16*, these animals do not have higher nuclear localization of DAF-16 [124]. Next, either knockout of or RNAi against *cep-1*, a transcription factor that regulates the germline response to DNA damage and is an ortholog of the tumor-suppressor p53, increases longevity in *C. elegans* in a *daf-16*-dependent manner, yet RNAi against *cep-1* does not alter DAF-16 nuclear localization [125]. Further, *cep-1* mutants, though long-lived, are not resistant to heat, oxidative, UV, or pathogenic stress [125]. A related result involves the conserved gene *smg-1*, which encodes a serine-threonine kinase that functions in nonsense-mediated mRNA decay. Inactivation of *smg-1* by mutation or RNAi confers increased lifespan and resistance to oxidative stress in a *daf-18-*, *daf-16-*, and *cep-1*-dependent manner, yet in these animals DAF-16 is not localized to the nucleus to any degree beyond that in controls [126]. Finally, nuclear localization of DAF-16 in wild-type *C. elegans* occurs in response to starvation, heat shock, and oxidation (by treatment with juglone), but very little or not at all in response to UV irradiation [124, 127].

The effect of the IIS pathway on *C. elegans* lifespan seems to involve communication between cells and between tissues. Some *daf-2* mosaic animals exhibit longer lifespans than wild-type, suggesting that *daf-2* acts cell nonautonomously to modulate *C. elegans* lifespan (if *daf-2* acted cell autonomously, then the *daf-2(+)* tissues of the mosaic would be expected to die earlier and thus kill the animal, resulting in a normal rather than a lengthened lifespan) [128]. One potential explanation for the cell-nonautonomy of *daf-2* is that the IIS pathway activates the expression of the insulin/IGF-1 homolog INS-7, which may potentially activate the IIS pathway in other cells [107]. Other potential signaling molecules regulated by DAF-2 and DAF-16, such as the putative secreted protein encoded by *scl-1*, may also contribute to the cell non-autonomy of *daf-2* [107]. Counterintuitively, tissue-specific studies suggest that DAF-2 and DAF-16 act in different tissues to influence *C. elegans* lifespan: DAF-2 functions mostly in the nervous system to modulate *C. elegans* longevity [129], whereas rescue of *daf-16* in the intestine is the most effective in restoring the lifespan and thermotolerance of *daf-2;daf-16* mutants [130]. To explain this discrepancy in tissue-of-action, it has been proposed that *daf-2(+)* neurons may produce a signal that decreases DAF-16 function in intestinal cells; however an assay using the *sod-3::gfp* reporter suggests that DAF-16 activity in these animals remains high in the intestine [130]. Experiments on this subject remain difficult to interpret.

A more recently identified target of the IIS pathway is SKN-1, a transcription factor that orchestrates the oxidative stress response in adult *C. elegans*, independent of DAF-16 activity, by activating the expression of genes coding for enzymes including catalases, SODs, and some glutathione *S*-transferases [131-133]. *skn-1* mutants are short-lived and exhibit sensitivity to oxidative stress, and overexpression of SKN-1 in intestinal cells increases lifespan and oxidative stress resistance [131-133]. Mutations in *skn-1* also suppress the longevity and oxidative stress resistance of *daf-2* mutants [131]. Recently, it has been shown that IIS directly inhibits SKN-1 via phosphorylation by AKT-1, AKT-2, and SGK-1, whereas in IIS mutants, SKN-1 accumulates in the nucleus of intestinal cells and activates expression of genes involved in the oxidative stress response [131, 132]. Consistent with SKN-1 regulation of stress resistance, RNAi of two genes upregulated by SKN-1, *nlp-7* and *cup-4*, leads to reduction in lifespan and oxidative stress resistance [134]. RNAi of *ent-1*, which is down-regulated by SKN-1, increases oxidative stress resistance without affecting longevity [134].

Many IIS pathway genes were initially identified in *C. elegans* based on their regulation of dauer formation. Dauer larvae are stress resistant, and because their growth is arrested they can have significantly lengthened lifespans, though nematodes that have exited dauer have a normal lifespan from that point on. In many respects, dauers and long-lived IIS mutant animals appear to have many similarities. However, the longevity of IIS pathway mutants can be separated from the effects on dauer formation. “Class 2” *daf-2* mutants exhibit dauer-like traits as adults, while “class 1” mutants do not, yet both classes of mutants have extended lifespan [75, 93]. Longevity extension and the appearance of dauer-like traits can be separated by manipulating the timing of *daf-2* expression, as demonstrated by an elegant work using RNAi against *daf-2* [135]. Initiating *daf-2*(RNAi) in adulthood confers essentially the same degree of longevity and stress resistance as initiating *daf-2*(RNAi) at hatching; conversely, knockdown of *daf-2* during development only does not have extended lifespan at 20°C, which suggests that the *daf-2* pathway acts during adulthood, but not during development, to influence adult lifespan [135]. Further, the temporal requirements of longevity by IIS are also separate from those of other longevity pathways, suggesting that these pathways increase lifespan independently: reductions in mitochondrial respiration only increase lifespan if enacted early in the nematode's life, while dietary restriction extends longevity regardless of the timing [123].

The IIS pathway mutants reflect a correlation between longevity and stress resistance. Many of the genes downstream of *daf-2* and *daf-16* have clear roles in stress resistance, although it is not as clear how they influence aging. However, inconsistencies exist in the correlation between longevity and stress resistance. Moreover, current results give only an incomplete understanding of their association, and further study of the lifespan and stress response of various IIS pathway mutants is necessary.

### MicroRNA regulation of aging

microRNAs (miRNAs) are small, noncoding RNA molecules that post-transcriptionally suppress gene expression by binding to their target mRNAs. They play important roles in many biological processes, including developmental timing, cancer, and aging. The miRNA *lin-4* and its target *lin-14*, which act in the heterochronic pathway to regulate the timing of *C. elegans* larval development, have also been found to modulate aging: reduced activity of *lin-4* accelerates tissue aging in *C. elegans*, whereas increased activity of *lin-4* or reduced activity of *lin-14* increases lifespan in a DAF-16- and HSF-1-dependent manner [89]. Further, this study found that the longevity of *daf-2* mutants depends on *lin-4*; thus, the modulation of lifespan by *lin-4* and *lin-14* is intimately linked to the IIS pathway. In addition, *mir-71*, *mir-239*, and *mir-246*, which each increase significantly in expression during aging in wild-type *C. elegans*, have longevity and stress-resistance phenotypes [136]. Deletions of *miR-71* and *miR-246* decrease nematode lifespan, and deletion of *miR-239* increases lifespan, while overexpression of these miRNAs has the opposite effects.

Furthermore, *miR-71* mutant animals are more sensitive to thermal and oxidative stress, whereas *mir-239* mutants exhibit increased resistance to these stressors. Deletion of *miR-71* partly suppresses the extended lifespan of nematodes treated with *daf-2*(RNAi), and *daf-16* fails to further shorten the lifespan of *miR-71* mutants; in addition, several members of the IIS pathway are putative targets of *miR-71*, further suggesting that the mechanism behind the lifespan effect of *miR-71* is related to the IIS pathway [136]. More recently, inter-individual fluctuations in the levels of reporters for each of these microRNAs were shown to quantitatively predict individual lifespan (within a genetically identical cohort) [137]. Of these, miR-71 reporter levels measured during early adulthood predict up to 47% of longevity variation; this predictive ability required *daf-16*, suggesting that fluctuations in wild-type IIS levels set lifespan in intact individuals. This confirms another recent finding that *sod-3*::GFP, a well-known reporter of IIS levels, also predicts individual longevity [138].

### Target of rapamycin pathway

Target of rapamycin (TOR) is an evolutionarily conserved serine/threonine protein kinase that promotes cell growth and metabolism in response to nutrients [139]. Under stress, TOR activity goes down, resulting in lower levels of protein synthesis and increased autophagy. The homolog of TOR in *C. elegans* is *let-363*, and that of the TOR binding partner Regulated Associated Protein of TOR (Raptor) is *daf-15* [140]. In nematodes, deficiency in *let-363* or *daf-15* results in developmental arrest, intestinal atrophy, and death as dauer-like larvae [140, 141]. Non-lethal loss of *let-363* or *daf-15* can be achieved with *daf-15* heterozygotes, *let-363*(RNAi) starting in adulthood, or mutations for decreased *let-363* activity, all of which result in lifespan extension [19, 140, 142]. In addition, *let-363*(RNAi) confers greater thermotolerance on nematodes [142]. The TOR pathway may also be linked to the IIS pathway, as DAF-16 has been found to control the expression of *daf-15* [140]. Moreover, TOR RNAi does not further extend the lifespan of *daf-2* mutants, whereas it does extend the lifespan of *daf-16* animals [143]. These mixed results suggest that the TOR pathway interacts with IIS in some fashion, but perhaps independently or downstream of *daf-16* [143].

The mechanism of TOR's influence on lifespan is similarly unclear. One possibility is that the TOR pathway influences lifespan by regulating mRNA translation. Mammalian TOR phosphorylates the translation initiation factor 4E binding protein 1 (4E-BP1), which reduces its affinity for the eukaryotic initiation factor 4E (eIF4E), thus allowing eIF4E to function in mRNA translation. In this way, TOR activity promotes mRNA translation. Similarly, *let-363* and *daf-15* mutant *C. elegans* exhibit decreased protein synthesis and generally bear similarities to deficiencies in mRNA translation initiation factors [139-141]. This suggests that *let-363* might function through a pathway analogous to that of mammalian TOR. However, there is no known homolog of 4E-BP1 in *C. elegans* [144]. Deletion of *ife-2*, the homolog of eIF4E, extends lifespan independently of *let-363*, and has also been shown to reduce protein synthesis and increase resistance to oxidative stress [144]. In addition, knockdown of TOR further increases lifespan in animals carrying an *ife-2* deletion, indicating that *ife-2* and the TOR pathway influence longevity through at least partly distinct mechanisms [144]. Even if *let-363* promotes longevity by decreasing protein synthesis, this cannot be the only mechanism of lifespan extension for the TOR pathway, since decreasing translation initiation factor levels by eIF2β or eIF4G RNAi, or by *ife-2* mutation, does not extend lifespan in *daf-16* mutants and actually shortens that of *daf-2* mutants, in contrast to the results of *let-363*(RNAi) [142]. Another possibility is that *let-363* extends lifespan by promoting autophagy: mutants with low TOR pathway activity have increased autophagy, and *bec-1*, which functions in the formation of autophagosomes, is required for the longevity of *daf-15* heterozygotes [145]. This makes an interesting parallel with the role of autophagy in lifespan extension by mutations in the IIS pathway, as *daf-2* mutants also have increased autophagy, and *bec-1* is also required for the longevity of *daf-2* mutants [94]. Once again, the TOR and IIS pathways appear to have some overlap, though the details of their mechanistic relationship remains ambiguous.

### Autophagy

Autophagy is a suite of cellular recycling processes in which macromolecules and organelles in the cell are degraded into raw materials that can then be used for the synthesis of new macromolecules. During autophagy, large vesicles full of cellular materials to be recycled, called autophagosomes, are formed and fuse with lysosomes, effecting the degradation of their contents. This process is typically held in check by TOR signaling, which is decreased through diverse signaling pathways in response to different stressors [146]. The protein LGG-1 is the *C. elegans* ortholog of the vacuolar protein Atg8/MAP-LC3, which is incorporated into autophagosome membranes, and GFP-tagged LGG-1 is frequently used as an indicator of autophagy [94]. Using this indicator, autophagy has been shown to be upregulated in nematodes under dietary restriction (DR) [145, 147], IIS inhibition [94], or TOR inhibition [145], as well as in animals that have entered the dauer state [94]. Both the longevity and increased autophagy of DR animals require the FOXA transcription factor PHA-4, suggesting that autophagy is a transcriptionally regulated response to DR [145, 148]. On the other hand, though the FOXO transcription factor DAF-16 is required for the longevity of IIS mutants, it is not necessary for the increased autophagy of *daf-2* animals [145]. Various autophagy genes have been shown to be required for the longevity of IIS and TOR mutants, as well as DR animals. RNAi knockdown of either of the autophagy genes *bec-1* and *atg-12* shortens the lifespan of *daf-2* mutants, while having a much smaller effect on the lifespan of wild-type animals [94, 149]. Mutants heterozygous for *daf-15* also require *bec-1* for their increased longevity, and both *bec-1* and *vps-34*, which encodes a phosphatidylinositol 3-kinase essential for autophagy, are required for the longevity of *eat-2* mutants, which are a genetic model for DR [145]. Autophagy appears to be an essential part of the longevity pathways activated in IIS mutants, TOR mutants, and DR animals. Given that autophagy is upregulated in response to stress (see also: [150]), the connection between autophagy and longevity is likely related to stress response pathways.

### Mitochondrial respiration

Mitochondrial respiration defects caused by mutation or RNAi can often extend *C. elegans* lifespan [8, 106, 109, 151-154], but the influence of these defects on stress resistance varies. For instance, *isp-1* encodes the iron sulfur protein of complex III in the electron-transport chain in *C. elegans*, and a mutation in this gene yields significantly increased lifespan and resistance against paraquat [8, 9]. However, though RNAi inactivation of components of the electron-transport chain often increases resistance to H_2_O_2_ and high temperatures, it also increases sensitivity to paraquat [152]. Similarly, RNAi against *cchl-1*, the *C. elegans* Cytochrome c heme lyase – an enzyme that covalently attaches heme to cytochrome c, which is essential to the electron-transport chain [155] – confers longer lifespan, but not resistance to paraquat or H_2_O_2_ [123, 152].

The mechanisms behind the extended lifespan of nematodes with mitochondrial dysfunction are not well understood. Since ROS are produced as byproducts of mitochondrial respiration, one possible explanation is that mutations in mitochondrial respiration genes reduce ROS production. In support of this hypothesis, the long-lived *isp-1* mutant has decreased oxygen consumption. The leucyl-tRNA synthetase gene *lrs-2* is needed for the proper translation of genes encoded by the mitochondrial genome; *lrs-2* mutations, which are thought to result in low electron-transport chain activity, extend *C. elegans* lifespan [106, 109, 152]. However, several observations controvert the theory that reduced ROS accounts for the longevity of mitochondrial mutants. For one, lifespan extension by mutations in mitochondrial respiration genes does not appear to correlate with decreases in lifelong oxidative stress as measured by whole-animal protein carbonyl content [156]. Moreover, at least one mitochondrial respiration mutant, *clk-1*, does not exhibit reduced respiration and has normal or slightly elevated levels of ATP, although it does have increased lifespan and reduced behavioral rates [123, 151, 154, 157]. (The *clk-1* gene encodes a hydroxylase necessary for the biosynthesis of ubiquinone, which shuttles electrons as part of the mitochondrial respiratory chain [158].) Furthermore, not all disabling mutations in mitochondrial respiration lengthen lifespan. Mutants of *mev-1*, which codes for a subunit of complex II, have reduced mitochondrial respiratory rates and a thermotolerance comparable to wild-type, but also have a 30% decrease in lifespan [104, 159]. However, *mev-1* mutants express only half as much SOD as wild-type and are hypersensitive to paraquat [160], which makes it difficult to clearly interpret lifespan results from this strain. Together, these results suggest that the longevity of *C. elegans* mitochondrial respiration mutants cannot be explained solely by lower rates of oxygen consumption and ROS production.

Given the many observations inconsistent with the theory of reduced ROS, alternative hypotheses have been put forth to explain the longevity of mitochondrial respiration mutants. It has been suggested that some mitochondrial mutations may slightly increase ROS levels, resulting in lifespan extension by a hormetic effect [106, 161, 162]. Another proposal is that reduced respiration early in life may stimulate a regulated response that subsequently extends *C. elegans* lifespan [75, 123, 154]. Both ideas hold that reduced respiration may cause a type of stress, and the response of the nematode's cells to this stress might have a life-extending effect. Consistent with this theory, inhibition of certain mitochondrial respiration genes by mutation or RNAi leads to increased expression of certain cell-protective and metabolic genes, and greater abundance of mitochondrial DNA [163]. Further, RNAi against the potential signaling genes *fstr-1* and *fstr-2* reduces lifespan, increases behavioral rates, and suppresses gene expression changes in *clk-1* mutants [163]. These results suggest that *fstr-1* and *fstr-2* may be part of a pathway that triggers a life-extending response to reduced respiration in *clk-1* mutants [163]. However, these genes are not required for the longevity of all other mitochondrial mutants [163].

In most cases, lifespan extension by mutations in mitochondrial respiration genes does not depend on *daf-2* and *daf-16*, and is therefore independent of the IIS pathway [8, 106, 109, 151, 152, 154]. However, *qm50*, a point mutation in *isp-1*, does not extend the lifespan of *daf-2* mutants, and its effect on lifespan is independent of *daf-16* [8, 75].

### Antioxidant defenses

As discussed previously, many longevity mutants are resistant to oxidative stress and have altered levels of antioxidant enzymes. Mutants for *age-1*, which have a 40-110% longer lifespan than wild-type, express elevated levels of catalase and SOD [14, 34, 95, 104], while *mev-1* mutants, with a 30% shorter lifespan than wild-type, are hypersensitive to paraquat and have only about half the normal amount of SOD [16, 104, 159]. Furthermore, studies have found that oxidative damage (measured by protein carbonyl content) was decreased in *age-1* mutants and increased in *mev-1* mutants relative to wild-type [16, 104]. The short-lived *daf-16* mutants also express lower levels of SODs, although these animals also have higher expression levels of *ctl-1* and *ctl-2* as compared to wild-type animals [104]. The long-lived *skn-1* mutant also expresses high levels of antioxidant enzymes [131-133], and in *clk-1* mutants there is a significant increase in detoxification enzymes including UDP-glycosyl transferases, glutathione *S*-transferases, and SOD-3 [163].

Like *age-1* mutants, *daf-2* mutants are long-lived, resistant to oxidative stress, and have increased SOD and catalase expression [14, 34, 72, 91]. However, the longevity of *daf-2* mutants is only partly suppressed by RNAi against *ctl-1* (encoding a cytoplasmic catalase), *ctl-2* (encoding a peroxisomal catalase and peroxidase), or *sod-3* (encoding an iron/manganese SOD predicted to be mitochondrial)[107]. Long lived strains with mutations in *isp-1*, *daf-2*, *daf-2;clk-1*, *daf-2;isp-1*, or *daf-2;isp-1;ctb-1* show increased levels of both SOD-1 and SOD-2 but exhibit no quantitative correlation between increased SOD levels and lifespan [164]. For example, *daf-2* and *daf-2;clk-1* mutants express the same levels of SODs, but *daf-2;clk-1* mutants have much longer lifespan [8, 157, 164].

Moreover, directly altering the expression of antioxidant enzymes does not have a consistent effect on lifespan. Wild-type *C. elegans* treated with *sod-1*(RNAi) are more sensitive to paraquat but have only slightly shortened lifespan [164]. Mutations in *sod-3*, *sod-4*, or *sod-5* do not affect lifespan, and loss of *sod-2* might even increase lifespan despite higher levels of oxidative damage [36, 165, 166]. Similarly, deletion of *ctl-1* does not shorten lifespan [36].

## STRESS, HORMESIS, AND LONGEVITY

Pre-treatment of many organisms, including *C. elegans*, with mild degrees of stress can prompt extended lifespan (induced lifespan extension), as well as greater resistance to oxidative stress (induced resistance to oxidative stress) and high temperatures (induced thermotolerance), in a process generally known as “hormesis”. [22, 48, 64, 65, 167]. The hormetic effect of thermal stress is dependent on members of the IIS pathway, though, interestingly, induced thermotolerance and lifespan extension have separate genetic requirements – induced thermotolerance depends on *daf-18*, while the lifespan effect is dependent on *daf-12*, *daf-16*, and *daf-18* [22, 167].

Several models have been proposed to explain how different levels of stress influence lifespan [25, 26, 64, 65]. Each is premised on the assumptions that during the aging process, random damage accumulates in the nematode. Exposure to stress leads to a response that includes a dramatic increase in the levels of HSP-4 and HSP-16 in *C. elegans*, and the levels of the heat-shock proteins correlate with the lifespan of the hormetically treated nematodes [58]. In the “stochastic” model, the accumulation of damage is proportional to the applied stress, but the production of HSPs is not; thus at certain doses, the HSPs produced by the cells of the nematode in response to stress are in fact more than necessary to repair the damage caused, and thus have long-term protective functions, increasing the nematode's longevity and tolerance to future stressors [24, 25, 58, 65]. The hormetic effect can also be explained in terms of energy capacity. In the “biphasic” model for survival, an organism's ability to restore its normal functioning after exposure to stress decreases linearly with age, and death occurs when its energy capacity becomes too low to overcome the damage caused by stress [64]. The model posits that a hormetic level of stress causes an organism to increase its energy capacity so that it can respond to the anticipated future stresses, resulting in slower aging and increased stress resistance [64]. It has also been proposed that an isogenic population of *C. elegans* is actually composed of subpopulations that each have a different mortality pattern (“discrete heterogeneity” model). Hormetic stress shifts nematodes from one subpopulation to another, having the overall effect of increasing mean lifespan [26]. These different models approach the problem of explaining hormesis from different angles. While they are not necessarily incompatible with one another, the mechanisms underlying the hormetic effect are nevertheless incompletely understood.

## DIETARY RESTRICTION

Dietary restriction (DR), which has been shown to prolong longevity across diverse taxa [168, 169], may also be considered a type of stress, as DR often leads to increased activities of antioxidant enzymes, as well as greater resistance to thermal and oxidative stress [19]. If no food is available when a *C. elegans* larva hatches, the nematode's growth is arrested as an L1 stage larva until food is provided, at which point the nematode grows normally and has a typical adult lifespan [170]. Similarly, dietary restriction and crowding of L1 or L2 larvae causes nematodes to enter the dauer stage [62]. DR can also lead to extended adult lifespan rather than prompting a state of arrested growth, but the degree of lifespan extension depends on the type of DR used. *C. elegans* can be subjected to DR by culturing in an axenic, completely-defined liquid medium or in lower concentrations of *E. coli* [106], by complete removal of bacterial food early in adulthood (“dietary deprivation”) [171-173], through mutants defective in feeding, such as the *eat* mutants which have limited pharyngeal pumping ability and thus limited food uptake, or via mutations in specific nutrient transporters [106]. Nematodes cultured in axenic medium have a two-fold increase in length of development and adult lifespan [174, 175]; adult *C. elegans* maintained on plates with lower amounts of bacteria have a 15-30% increase in lifespan [176]; and *C. elegans* with *eat* mutations or subjected to dietary deprivation early in adulthood, have lifespans lengthened by up to 50% [171, 172, 177]. If DR is a form of mild stress, lifespan extension through DR could be considered a type of hormesis [19, 23].

However, each method of DR suffers from potential confounding factors, making results difficult to interpret [19]. Since proliferating *E. coli* are associated in some fashion with early mortality in *C. elegans* [61], restricting the nematode's diet may lengthen lifespan by exposing *C. elegans* to fewer (or no) live bacteria rather than by the effect of decreased caloric intake. Further, restricting the *C. elegans* diet by lowering the concentration of *E. coli* or by *eat* mutation not only reduces calorie consumption but also limits intake of essential nutrients provided by the *E. coli* [19]. Likewise, though axenic medium contains all the compounds essential to *C. elegans*, it may still cause malnutrition – for instance, it may be more difficult for the nematodes to metabolize the compounds in the axenic medium, or to use the available compounds to synthesize a nutrient that is more directly available from *E. coli* [19, 178].

The physiological outcomes from dietary restriction can be diverse depending on the specific type of DR used. Mutants for *eat-2*, as well as wild-type grown in axenic medium or in lower concentrations of bacteria, have higher activities of SOD and catalase [174, 179]. *C. elegans* cultured on plates with lower amounts of bacteria or subjected to dietary deprivation in early adulthood have increased resistance to paraquat [172, 176], but nematodes grown in liquid culture with lower concentrations of bacteria are not more resistant to paraquat and H_2_O_2_ [179]. Growing wild-type or *eat-2* mutant nematodes in axenic culture results in greater resistance to high temperature, while *eat-2* mutants grown on bacterial lawns do not have increased thermotolerance [174]. Nematodes subjected to dietary deprivation in early adulthood also have greater thermotolerance [171, 172]. Interestingly, mutants in *pep-2*, which codes for a carrier responsible for the uptake of peptides, have greater tolerance to thermal and oxidative stress, but are not long-lived [19, 180]. It has been suggested that DR might extend lifespan by decreasing metabolic rate and ROS production [177]. However, studies of oxygen consumption and heat production have shown that nematodes cultured in axenic medium or in lower concentrations of bacteria do not have a reduced metabolism. Meanwhile, *eat-2* mutants grown in liquid culture actually have an increased metabolic rate, and the metabolic rate of *eat-2* mutants grown on solid medium has not been evaluated [174, 179].

Although DR is known to increase lifespan in many different species [168, 181], the mechanism behind DR-mediated longevity remains unknown. Given that *C. elegans* undergoing DR are both long-lived and resistant to thermal and oxidative stress, it is conceivable that DR results in the upregulation of stress defense systems that in turn increase lifespan as well as stress resistance, possibly through a hormetic effect [19, 181]. Higher activity of SOD and catalase has been found in *eat-2* mutants, in nematodes grown in axenic medium, and in nematodes cultured in lower concentrations of bacteria [174, 179]. Additionally, the longevity of DR animals requires the action of SKN-1 in the two ASI neurons [182], which control entry into dauer and are involved in chemotaxis to lysine [183]. As the transcription factor SKN-1 is known to stimulate the oxidative stress response [132], it has been suggested that the ASI neurons might detect the effects of dietary restriction and consequently activate *skn-1*, as well as trigger hormonal signals that lead to longevity [182]. However, knockout of SOD enzymes in DR nematodes does not significantly affect lifespan, though this result does not rule out the possibility that other aspects of the oxidative stress response may play a role in DR-mediated lifespan extension [184].

Because of the close connection between diet and the insulin/IGF-1 signaling pathway, one might expect the longevity effect of DR to be mediated by the IIS pathway [19]. However, life extension and increased stress resistance resulting from DR are still observed in *daf-16*, *age-1*, and *daf-2* nematodes [19]. These tests are not entirely conclusive because the *age-1* and *daf-2* alleles used for lifespan studies are reduction-of-function rather than null mutations. The *daf-16* allele, however, is a null. These results imply that the longevity effect of DR in *C. elegans* is either not dependent or only partially dependent on the IIS pathway, contrary to the result in flies and rodents, in which IIS plays a role in DR [185-187]. Another pathway that may be thought to mediate the longevity effect of DR is the TOR pathway, which responds to nutrient level. Indeed, DR does not further extend lifespan of *let-363* mutants, suggesting that DR lengthens lifespan by downregulating *let-363* [180]. However, this cannot be the only mechanism of DR-induced lifespan extension, since lifespan extension in *let-363* mutants is dependent on the IIS pathway [19]. Interestingly, subjecting dietary-restricted *C. elegans* to *let-363*(RNAi) also reduces the translation rate by an additional 49% without lengthening lifespan [142], indicating that TOR signaling and protein translation remains active even in DR, though perhaps at levels below those which manipulate longevity. The longevity effect of DR also correlates with the upregulation of autophagy: nematodes under DR have higher levels of autophagy [145, 147], and the autophagy genes *bec-1* and *vps-34* are required for the increased lifespan of *eat-2* mutants [145]. It has been suggested that autophagy is an essential part of longevity pathways related to nutrition in that it provides the raw materials needed for the synthesis of proteins that confer longevity and stress resistance [145].

## THEORIES OF AGING AND STRESS

Though longevity and stress resistance seem to be correlated, the mechanism behind such a correlation remains unclear. In this section we examine several theories of aging, which posit different, though non-exclusive, explanations for why longevity and stress resistance should be correlated.

### Free radical theory of aging

The free radical theory of aging (FRTA), proposed by Denham Harman in the 1950's, postulates that the aging of all living beings is caused by the common biochemical process of accumulation of oxidative damage over time [188, 189]. To protect against internal and external sources of ROS, the cell uses enzymes such as SOD, catalase, and glutathione peroxidase. According to FRTA, these anti-damage defenses are insufficient to eliminate all oxidative damage from the cell, or they might even lose efficiency over time, resulting in an accumulation of cell damage that constitutes aging [188].

Consistent with FRTA, longevity is often correlated with oxidative stress resistance and levels of antioxidant enzymes [14, 16, 34, 104, 159]. However, some longevity mutants are not resistant to oxidative stress, and levels of catalase and SOD do not always correlate with longevity in a quantitative manner [125, 152, 164]. Since most ROS are produced by mitochondria, changes in mitochondrial function might be expected to modulate lifespan [123]. Many, but not all, mitochondrial respiration mutants exhibit extended lifespan [8, 9, 104, 152]. Additionally, the inactivation of genes in mitochondrial respiration pathways must occur during development in order to affect lifespan, whereas based on FRTA the suppression of these genes in adult nematodes should also influence lifespan [123, 154].

The best way to test FRTA is to manipulate the level of oxidative damage and observe the effects on aging [36, 106]. FRTA is supported by evidence that *C. elegans* lifespan can be extended by the introduction of antioxidant compounds, but these results are not always reproducible, and it has been shown that some antioxidant molecules do not lengthen lifespan [36, 38, 40, 41, 46]. Moreover, changes in expression of antioxidant enzymes often do not have the expected effect on lifespan [36, 164-166]. Skeptics also argue that FRTA does not take into account the beneficial role of free radicals in normal functioning and survival [190]. Further, FRTA suggests that species with longer lifespans should be those with more efficient mechanisms for countering free radicals, but there is as yet no evidence that this is the case [190].

### Antagonistic pleiotropy

Absent social, altruistic/kin-selective, or “grandparenting” behaviors, post-reproductive traits typically cannot be selected for or against by evolution. As aging is largely a post-reproductive process, any genetically-controlled, stereotyped features of senescence may simply be by-products of adaptive traits that manifest earlier. Specifically, George Williams noticed that evolution favors genes that have a positive effect during development and the reproductive stage, regardless of any potential negative pleiotropic effects at post-reproductive ages [191, 192]. Therefore, alleles with positive effects until the reproductive phase, but negative effects at post-reproductive ages, are not selected against [191]. This observation is the foundation for Williams' theory of antagonistic pleiotropy [191], which predicts that stress response mechanisms and other pathways that benefit the organism would be activated early in life at levels ensuring optimal reproductive fitness, but reductions in activity at post-reproductive ages (“gene expression drift”) would not be selected against.

Several examples of antagonistic pleiotropy have been found in mammals [193-195], and the GATA transcription factor ELT-3 has been proposed as an example in *C. elegans* [196]. ELT-3 functions in hypodermal development, but also decreases in expression during normal aging [196, 197]. Since *elt-3* regulates the expression of *sod-3* and is required for the longevity of *daf-2* and *eat-2* mutants, it is conceivable that *elt-3* functions in a stress response pathway downstream of the IIS and DR-mediated longevity pathways [196, 198]. Expression of *elt-3* is negatively regulated by the GATA transcription factors ELT-5 and ELT-6, and these three genes may constitute an example of drift in a developmental pathway leading to the downregulation of a stress response pathway [196, 198, 199]. The role of the miRNA *lin-4* and its target *lin-14* on longevity and stress response may also be an example of antagonistic pleiotropy, given that *lin-4* and *lin-14* have known functions in *C. elegans* larval development [89].

### Disposable soma

Like antagonistic pleiotropy, the disposable soma theory centers on the relationship between longevity and fitness. This theory proposes that, since resources are limited, an organism must carefully allocate resources between reproduction and somatic maintenance, depending the animal's particular environment [106, 192, 200]. As stress responses may be particularly resource-intensive, this theory predicts that these responses will only be activated to the degree that will optimize an organism's reproductive fitness. Additional resource expenditure to optimize somatic fitness (i.e. lifespan) would not be selected for. As an example, the disposable soma theory also offers an explanation for lifespan extension by DR: when food is scarce, it is beneficial for the animal to use more energy for somatic maintenance and less for reproduction [106]. This might allow the animal to live long enough to be able to reproduce after conditions improve, in an environment more congenial to the success of its offspring [106, 201, 202]. More generally, stressful conditions of any type might shift the evolutionarily optimal resource allocation toward somatic maintenance, so that the animal might “live to reproduce another day”; any collateral lifespan extension this might induce beyond the reproductive period, however, would be evolutionarily neutral.

Since the disposable soma theory predicts a trade-off between reproduction and somatic maintenance, long-lived and stress resistant *C. elegans* might be expected to have reduced fertility. However, studies on the fertility of longevity mutants give mixed results [12, 93, 98]. The long-lived, stress-resistant *daf-2* mutant exhibits normal fertility levels, although it has a significant reduction in early fertility [93, 203], and the fertility of the long-lived *age-1* mutant is not significantly different from wild-type [12, 204, 205]. There is evidence that *pep-2* mutants, which are resistant to thermal and oxidative stress but are not long-lived, have reduced fertility under certain dietary conditions, but the broader relationship between stress resistance and fertility remains unclear [206]. Additionally, increasing production of eggs in *C. elegans* does not lead to a shorter lifespan, and the effect of such an increase on stress resistance has not been determined [207].

Fertility, especially as measured in lab conditions, is only one component of reproductive fitness, however. Various mutations conferring longevity and stress resistance have clear impact on fitness: *daf-2* mutants have significantly reduced early fertility and are outcompeted by wild-type within four generations [93, 203], while *age-1* mutants have lower fitness than wild-type under nutritional stress [204].

In further support of the disposable soma theory, it has been observed that removal of the germline extends lifespan and increases *C. elegans* resistance to thermal and oxidative stress [20, 21]. This suggests that resources can be devoted to somatic maintenance when not used by the reproductive system, and that when the germline is present, it down-regulates somatic stress responses, perhaps in order to conserve resources. The reproductive system of *C. elegans* consists of two parts – the germ cells and the somatic reproductive tissues. Removal of the entire reproductive system by laser ablation does not extend nematode lifespan; however, if the germline is removed and the somatic gonad left in place, *C. elegans* have increased thermal and oxidative stress resistance, as well as a ~60% lifespan extension [20, 21]. Additionally, both *glp-1* mutants, in which the germline does not proliferate, and *mes-1* mutants, which lack germ cells, are long-lived [21]. Thus, the somatic gonad is necessary for lifespan extension by germline removal; however certain stress-resistant phenotypes produced by DAF-16 activity in response to gonad loss do not require the somatic gonad [208]. This suggests that germline removal influences lifespan and stress resistance through at least partially separate mechanisms.

Lifespan extension by germline removal also requires *daf-16* [20, 91, 130]. However, *daf-16* is not necessary for germline removal to increase *C. elegans* resistance to paraquat and heat [130, 208]. This result again suggests that the longevity and stress resistance phenotypes of nematodes lacking a germline are conferred by distinct pathways. The signaling that links the germline to stress response mechanisms remains unclear. However, it has been observed that DAF-16 accumulates in the nuclei of intestinal cells in nematodes in which the germline has been removed [83, 208]. The effect of germline ablation on lifespan also depends on *kri-1*, the gene for an intestinal ankyrin-repeat protein; *daf-9*, the gene for a cytochrome P450 protein; and *daf-12*, which codes for a nuclear hormone receptor [99]. Interestingly, lifespan extension through the IIS pathway does not depend on these genes, suggesting that *kri-1*, *daf-9*, and *daf-12* function specifically in incorporating information on the status of the germline into the regulation of lifespan. Similarly, the predicted transcription elongation factor *tcer-1* is required for the lifespan effect of germline removal, but not for the effect of DR, decreased IIS, or *isp-1* mutation [209]. Furthermore, the *tcer-1* gene is required for the expression of many DAF-16 target genes upon germline removal, suggesting that *tcer-1* specifically links *daf-16* to longevity signals from reproductive tissues [209].

The longevity effect of germline removal is also related to the effects of dietary restriction. Lifespan extension by DR is not suppressed by removal of the entire reproductive system, but it is suppressed when only the germ cells are removed [210]. This suggests that germline removal and DR might activate converging pathways in lifespan extension.

## DISCUSSION

Studies on *C. elegans* have revealed a close connection between stress and aging. Although most long-lived mutant nematodes also exhibit increased stress resistance, exceptions and inconsistencies remain. Genes in the insulin/IGF-1 signaling pathway and in the target of rapamycin pathway, as well as those coding for mitochondrial respiration proteins and antioxidant enzymes, seem to play critical roles in both modulating stress response and aging. However, the mechanisms behind many of these modulations, and of the precise relationship between stress responses and aging, remain unclear. In Table 1, we attempt to summarize the genes that have been identified to play a role in aging and/or stress responses, highlighting cases where the two do not clearly overlap.

The role of free radicals in aging is supported by correlation between longevity, oxidative stress resistance, and elevated levels of antioxidant enzymes. However, this correlation is incomplete, and studies modulating levels of reactive oxygen species have obtained mixed results. The theory of antagonistic pleiotropy explains the modulation of longevity from an evolutionary point of view, and the few extant examples of antagonistic pleiotropy in *C. elegans* have possible connections to stress responses. The disposable soma theory is supported by the longevity of germline-ablated nematodes, and genes have been identified that seem to carry signals between the reproductive tissues and both aging and stress response mechanisms. These theories are of course not mutually exclusive, and, indeed, overlap to a large degree (and make similar predictions) regarding the relationship between stress responses, evolutionary fitness, and longevity. Both aging and stress responses are complex, multi-factorial processes that integrate internal and external signals over long time-spans, and it is likely that no simple theoretical explanation or set of experimental findings will fully predict or explain the many connections between the two.
